# Endoplasmic reticulum stress induces spatial memory deficits by activating GSK‐3

**DOI:** 10.1111/jcmm.13626

**Published:** 2018-04-19

**Authors:** Li Lin, Jie Cao, Shu‐Sheng Yang, Zheng‐Qi Fu, Peng Zeng, Jiang Chu, Lin‐Na Ning, Teng Zhang, Yan Shi, Qing Tian, Xin‐Wen Zhou, Jian‐Zhi Wang

**Affiliations:** ^1^ Department of Pathophysiology School of Basic Medicine and the Collaborative Innovation Center for Brain Science Key Laboratory of Ministry of Education of China and Hubei Province for Neurological Disorders Tongji Medical College Huazhong University of Science and Technology Wuhan China; ^2^ Cell Molecular Biology Laboratory of Basic Medical College Hubei University of Chinese Medicine Wuhan China; ^3^ Department of Traditional Chinese Medicine Wuhan Red Cross Hospital Wuhan China; ^4^ Co‐innovation Center of Neuroregeneration Nantong University Nantong China; ^5^ Present address: Department of Pathology and Pathophysiology School of Medicine Jianghan University Wuhan China

**Keywords:** Alzheimer's disease, cAMP response element binding protein, endoplasmic reticulum, glycogen synthase kinase‐3, spatial memory deficits

## Abstract

Endoplasmic reticulum (ER) stress is involved in Alzheimer's disease (AD), but the mechanism is not fully understood. Here, we injected tunicamycin (TM), a recognized ER stress inducer, into the brain ventricle of Sprague‐Dawley (SD) rats to induce the unfolded protein response (UPR), demonstrated by the enhanced phosphorylation of pancreatic ER kinase (PERK), inositol‐requiring enzyme‐1 (IRE‐1) and activating transcription factor‐6 (ATF‐6). We observed that UPR induced spatial memory deficits and impairments of synaptic plasticity in the rats. After TM treatment, GSK‐3β was activated and phosphorylation of cAMP response element binding protein at Ser129 (pS129‐CREB) was increased with an increased nuclear co‐localization of pY126‐GSK‐3β and pS129‐CREB. Simultaneous inhibition of GSK‐3β by hippocampal infusion of SB216763 (SB) attenuated TM‐induced UPR and spatial memory impairment with restoration of pS129‐CREB and synaptic plasticity. We concluded that UPR induces AD‐like spatial memory deficits with mechanisms involving GSK‐3β/pS129‐CREB pathway.

## 1 INTRODUCTION

Alzheimer's disease (AD) is a chronic neurodegenerative disorder with progressive impairment of memory and other cognitive functions. Apart from the loss of synapses, AD is histopathologically characterized by the accumulation of numerous intracellular neurofibrillary tangles (NFTs) and extracellular plaques.^1^, ^2^ Previous studies have shown that the amount of NFTs, mainly composed of the hyperphosphorylated tau,^3^, ^4^ in the AD brains is correlated with the degree of dementia.^5^, ^6^ Studies also showed that β‐amyloid (Aβ), forming toxic oligomers that aggregate into amyloid plaques, was associated with age‐related memory impairment.^7^, ^8^ Our previous studies indicate that tunicamycin (TM) could induce AD‐like tau hyperphosphorylation and reduction in some synapse‐related proteins in temporal cortex, frontal cortex and hippocampus.^9^ However, whether TM treatment affects learning and memory and the molecular mechanisms are unknown.

Endoplasmic reticulum (ER) is an important cellular organelle, responsible for the posttranslational processing of newly synthesized proteins and ensuring proper protein folding and assembly.^10^ ER stress is an important form of ER dysfunction, and ER stress has been observed in several neurological conditions, such as AD, Parkinson's disease, Amyotrophic lateral sclerosis and so on. Some studies have showed neurons are constantly exposed to ER stress in the AD brains. ER stress could be expressed by chaperone proteins and trigger many rescuer responses, including unfolded protein response (UPR) and ER‐associated degradation.^11^, ^12^, ^13^ The ER chaperone binding immunoglobulin protein (Bip) is physiologically bound to 3 important proteins in the ER membrane, pancreatic ER kinase (PERK), inositol‐requiring enzyme‐1 (IRE‐1) and activating transcription factor‐6 (ATF‐6). When UPR is induced, Bip is attracted to bind to the unfolded proteins accumulated in the ER to keep the correct protein folding and is thereby released from PERK, IRE‐1 and ATF‐6, which are consequently phosphorylated and activated.^14^, ^15^, ^16^ Although the initial UPR protects the cell from the toxicity of misfolded proteins in the ER, prolonged UPR activation may participate in the pathogenesis of protein misfolding diseases, such as AD.^17^, ^18^, ^19^, ^20^

Recently, several reports have shown that the UPR is activated in the AD brain. Bip, an ER stress marker, is increased in the temporal cortex and the hippocampus of AD cases compared with no demented control cases.^21^ The phosphorylated PERK (pPERK), an UPR activation marker, is most abundant in neurons with diffuse localization of the phosphorylated tau protein in the brain of AD patients.^22^ Our previous report has also shown that TM treatment induces tau hyperphosphorylation in frontal cortex, temporal cortex and hippocampus in rats with an increased level of Bip and reduction in some synapse‐related proteins,^9^ while overexpressing SIL1 rescued Bip elevation‐related Tau hyperphosphorylation in ER stress.^23^

Glycogen synthase kinase‐3 (GSK‐3) is highly expressed in the central nervous system (CNS) 24 and plays an important role in AD. Studies showed that the activated form of GSK‐3 was elevated in the AD brains.^25^ GSK‐3 could not only phosphorylate tau at most of the AD sites 26, 27, 28 but also induce Aβ overproduction.^29^ Activation or overexpression of GSK‐3 induces memory deficit,^30^, ^31^, ^32^ whereas inhibition of GSK‐3 reverses this effect.^31^, ^33^ However, the mechanism by which GSK‐3 regulates learning and memory is only partly understood. Recent study has found that overexpression of GSK‐3β could cause memory deficits by inhibiting long‐term potentiation which is accompanied by prominent impairment of synapses.^34^, ^35^ An in vitro study also show that GSK‐3β could be activated during ER stress 36, 37 and induce tau hyperphosphorylation 23, 38 that be involved in memory impairment. However, it is still not understood whether and how GSK‐3β plays an in vivo role in ER stress‐induced spatial cognitive alterations.

The cAMP response element binding protein (CREB), named by Montminy,^39^ regulates transcription of multiple genes in eukaryotic nuclei. CREB plays an important role in increasing long‐term potentiation (LTP), synaptic plasticity, development, differentiation and survival of neurons. Phosphorylation of CREB at Ser133 increases CREB activity, whereas phosphorylation at Ser129 and Ser142 inhibits its activity. GSK‐3β is an important kinase regulating the transcription of CREB.^40^

In this study, we established a rat model with activated UPR by brain injection of TM. We found that TM infusion induces spatial memory deficits in rats with ER stresses, shown by the increased level of phosphorylated PERK, IRE‐1, ATF6, CREB at Ser129, GSK‐3β at Tyr216 and impairment of synapses. Simultaneous inhibition of GSK‐3 rescues the UPR‐induced spatial memory impairments with restoration of ER stress and the associated dysfunction.

## 2 MATERIALS AND METHODS

### 2.1 Antibodies and chemicals

The antibodies used in this study are listed in Table 1. TM was from Alexis Biochemical (San Diego, CA, USA). TM was dissolved in DMSO at concentration of 12.5 mmol/L and stored at −20°C. SB216763 (SB) was from Tocris Bioscience (Bristol, UK) and freshly dissolved in DMSO from light before use. Bicinchoninic acid (BCA) protein detection kit was from Pierce Chemical Company (Rockford, IL, USA). Enhanced chemiluminescence was from Santa Cruz Biotechnology, Inc (Santa Cruz, CA, USA).

**Table 1:** A list of antibodies and their epitopes on the molecule of protein used in this study

Antibody	Epitopes	Type	Dilution	Source
GSK‐3β	Total‐GSK‐3β	Poly‐	1:1000 for WB	Cell Signalling (Danvers, MA, USA)
pS9‐GSK‐3β	Phospho‐GSK‐3β at Ser9	Poly‐	1:1000 for WB	Cell Signalling (Danvers, MA, USA)
Tyr216‐GSK‐3β	Phospho‐GSK‐3β at Tyr279/Tyr216	Mono‐	1:1000 for WB 1:200 for IFC	Millipore (Billerica, MA, USA)
PERK	Total PERK	Poly‐	1:1000 for WB	Cell Signalling (Danvers, MA, USA)
pPERK	Phospho‐PERK(Thr980)	Poly‐	1:1000 for WB	Cell Signalling (Danvers, MA, USA)
IRE1	Total IRE1	Poly‐	1:1000 for WB	Abcam (Cambridge, UK)
P‐IRE1	Phospho‐IRE1(Ser724)	Poly‐	1:1000 for WB	Abcam (Cambridge, UK)
ATF6	Total ATF6	Poly‐	1:1000 for WB	Abcam (Cambridge, UK)
P‐ATF6	Phospho‐ATF6	Poly‐	1:1000 for WB	Abcam (Cambridge, UK)
NR2A	Total NR2A	Poly‐	1:1000 for WB	Abcam (Cambridge, UK)
NR2B	Total NR2B	Poly‐	1:1000 for WB	Abcam (Cambridge, UK)
PSD95	Total PSD95	Poly‐	1:1000 for WB	Cell Signalling (Danvers, MA, USA)
Synapsin 1	Total Synapsin 1	Poly‐	1:1000 for WB	Millipore (Billerica, MA, USA)
CREB	Total CREB	Poly‐	1:1000 for WB	Cell Signalling (Danvers, MA, USA)
pS129‐CREB	Phospho‐CREB at Ser129	Poly‐	1:1000 for WB 1:200 for IHC 1:200 for IFC	Sigma (NY, USA)
pS133‐CREB	Phospho‐CREB at Ser133	Poly‐	1:1000 for WB 1:200 for IHC 1:200 for IFC	Cell Signalling (Danvers, MA, USA)
GAPDH	Full‐length GDPDH	Mono‐	1:1000 for WB	Abcam (Cambridge, UK)
Histone 3 (H3)	Total histone 3 protein	Poly‐	1:1000 for WB	Cell Signalling (Danvers, MA, USA)
DM1A	Alpha‐tublin	Mono‐	1:2000 for WB	Abcam (Cambridge, UK)

IHC, immunohistochemistry staining; IFC, immunofluorescence staining; Mono‐, monoclonal; Poly‐, polyclonal; WB, Western blotting.

### 2.2 Drug administration

Three‐month‐old (250 ± 20 g) male Sprague‐Dawley rats were supplied by the Experimental Animal Central of Tongji Medical College. All experimental procedures were approved by the Animal Care and Use Committee at the Huazhong University of Science and Technology and were performed in compliance with National Institutes of Health guidelines on the ethical use of animals. Rats were kept in cages under a 12‐hour light: 12‐hour dark (L/D) cycle with the light on from 7:00 am to 7:00 pm.

The rats (rats for each group were used in this study are listed in Table 2) were anaesthetized with 6% chloral hydrate (400 mg/kg) and placed in a Jiangwan‐II stereotaxic instrument (Jiangwan Medical Instrument Co. Shanghai, China).^41^ The skull was cleaned, and the hole (diameter 1.0 mm) was made for the infusion after the scalp was incised (5.0‐8.0 mm). For the lateral ventricular infusion, the coordinate of AP‐0.8, L‐1.5, V‐4.0 (in mm from bregma and dura, flat skull) was selected according to the stereotaxic atlas of Franklin and Paxinos. A sterilized needle connected to a Hamilton syringe was used to deliver TM or in combination with SB into the lateral ventricle (10 μL). Equal volume of DMSO with 0.9% NaCl was infused as vehicle controls.

**Table 2:** A list of rats used in this study

Groups	Nor	DMSO		TM (μm)						TM + SB	
				25		50		75			
		24 h	48 h	24 h	48 h	24 h	48 h	24 h	48 h	24 h	48 h
Western blotting	6	6	6	6	6	6	6	6	6	6	6
Behavioural test		20	20^a^			20	20^a^			20	20^a^

a Six rats of the group were used for Nissl and Immunofluorescence staining, 3 other rats were used for Golgi staining.

### 2.3 Behavioural test

Spatial memory was measured by Morris water maze test.^42^ The Morris water maze apparatus was the same as previously described.^43^ In brief, it consisted of a circular pool, 150 cm in diameter and 50 cm in height, with the interior painted black. The escape platform was made of clear plexiglass, 14 cm in diameter and 27 cm in height and was located 1 cm below the surface of the water. Visual cues were visible to the rats, including several geometric shapes that measured at least 20 cm in height and were positioned so that they were 15 cm above water level and remained distal and constant to the rats at all times. The water was maintained at 24 ± 2°C and was made opaque by the addition of non‐toxic black ink that generated the obvious contrast with the white skin of rats to record their movements. The swimming pathways and the latencies of the rats to find the hidden platform were recorded each day by a video camera fixed to the ceiling of the room, 1.5 m from the water surface. The camera was connected to a digital‐tracking device attached to an IBM computer loaded with the water maze software. The less time a rat spent in finding the platform, the better it scored the spatial learning and memory.

For spatial learning, rats were trained in water maze to remember the hidden platform for 7 consecutive days. For the first day, the rats only proceeded with one trial that started from the first quadrant. And for the following 5 days, 3 trials were performed for each rat every day, starting from the first, the second and the fourth quadrant, respectively. For the last training day (the 7 day), only one trial that started from the first quadrant's movements were performed, and total 20 trials were finished for 7 days’ training. On each trial, the rat started from each quadrant by facing the wall of the pool and ended when the animal climbed on the platform. The rats were not allowed to search for the platform more than 60 seconds, after which they were guided to the platform. Through these training sessions, rats acquired spatial memory about location of the safe platform, and rats that could find the platform after training from quadrant 1‐4 in turn for 24 trials within 20 seconds were selected and randomly divided into 3 groups for the brain lateral ventricle injections, respectively, with DMSO (10 μL) or TM (50 μmol/L, 10 μL) or TM + SB (50 μmol/L, 10 μL) as mentioned above. At 24 and 48 hours after the injection, the spatial memory retention of the rats was tested in the same water maze with the searching time extended to 90 seconds. After the behaviour test, the rats were killed for the rest studies.

Memory was also measured by step‐down avoidance tests which was made of electrically conductive metal fence at the bottom, around with open transparent plastic box (length × width × height: 23 × 23 × 40 cm). A wooden platform was placed on the bottom of the metal fence (length × width × height: 3.5 × 3.5 × 2.5 cm). Put the rats into the experimental device to make them be familiar with the surrounding environment for 5 minutes, respectively. And then began the stage of learning to put the rats gently on the platform, when animals jumping off the platform to the wire fence, immediately gave them electric shock (0.5 mA, for 5 seconds). After that put them back to the platform and began to record duration, if the duration reached 60 seconds that indicated rats have learned how to avoid electric shock. If the duration on the platform was <60 seconds, gave the rats electric shock again when they jumped off the platform until they could stay on the platform at least 60 seconds. Record the number of rats received shocks (mistake number) and the total time (learning time for the first time), the whole period was 5 minutes. The laboratory equipment should be cleaned with 75% alcohol after each rat was tested. When all rats were trained, following start the formal test to check the memory retention at 24 and 48 hours after DMSO or TM being injected. The rats were put on platform in this phase experiment, and the duration period and numbers of wrong were recorded within 5 minutes.

### 2.4 Cytoplasm–nucleus protein extraction

The 250‐mg fresh rats brain or cryopreserved tissue in −80°C were taken immediately into the glass homogenizer with ice pre‐cooling, add 500 μL cytosol extraction reagent (CER, the proportion of brain tissue with CER is 1:2) in it. Triturate the brain tissue with pestle and then fluctuate homogenate them by manual operation for 20 times. Ice bath for 10 minutes and then fluctuate homogenate them for 7 times again. Take 500‐μL lysate, transfer it to the new centrifugal tube, centrifuge for 5 minutes with 800 *g* in 4°C circumstance. The crude product of nucleus precipitates at the end of the pipe and the supernatant is the mixture of cell membrane and cytoplasm. Transfer the supernatant to the new centrifugal tube and add membrane extraction reagent (MER, 1/10 volume of the supernatant fluid) into it. Ice bath for 5 minutes, centrifuge for 30 minutes with 10956.4 *g* in 4°C circumstance. Take the supernatant into the new centrifugal tube which is the cytoplasm components and the precipitation is cell membrane components including cell membranes and organelles fragments which can be suspended again with 50‐100 μL suspension buffer. Add 500 μL nuclear extraction reagent (NER) into the crude product of nucleus obtained such as the above and vibrate it to suspend again. 4000 *g*, 4°C centrifuge for 5 minutes, abandon the supernatant and then add 500 μL NER into it to suspend it again. Repeat the above centrifugal steps and clear the centrifugal supernatant. Add 50‐100 μL suspension buffer to suspend the precipitation again to obtain the nuclei.

### 2.5 Western blotting

For brain samples, the hippocampus taken immediately after Morris water maze test were homogenized in buffer containing 10 mmol/L Tris–Cl, pH 7.6, 50 mmol/L NaF, 1 mmol/L Na3VO4, 1 mmol/L edetic acid, 1 mmol/L benzamidine, 1 mmol/L PMSF and a mixture of aprotinin, leupeptin and pepstatin A (10 μg/mL each) or obtain the cytoplasm–nucleus protein extraction as the above way. Three volumes of the homogenate for brain samples were added to one volume of the extracting buffer containing 200 mmol/L Tris–Cl, pH 7.6, 8% SDS, 40% glycerol, and the samples were boiled in water bath for 10 minutes and then followed by sonication for 15 seconds on ice. After measurement of protein concentration in the extracts using BCA kit (Pierce, Rockford, IL), a final concentration of 10% β‐mercaptoethanol and 0.05% bromophenol blue was added. The proteins in the extracts were separated by 10% SDS‐PAGE and transferred to nitrocellulose membrane. The membranes were blocked with 5% non‐fat milk dissolved in TBS‐Tween‐20 (50 mmol/L Tris–HCl, pH 7.6, 150 mmol/L NaCl, 0.2% Tween‐20) for 1 hour and probed with primary antibody (see Table 1 for detail) at 4°for overnight. Then, the blots were incubated with anti‐mouse or anti‐rabbit IgG conjugated to horseradish peroxidase (1:15 000) for 1 hour at room temperature and scanned after being washed with TBS‐Tween‐20,and the greyscale was analysed with odyssey system.

### 2.6 Nissl staining

Nissl staining was established in 1892 by Franna Nissl, the German pathologist, with alkaline dye to discover Nissl body, and this method was widely used. The picked brain slices were pasted on the glass slide disposed by gelatine in PBS liquid, dustproof atmospheric drying. And then, 1% Toluidine blue was dropped on the brain slices, keeping 5‐10 minutes, 95% alcohol was used to differentiate and observed under a microscope at the same time until the background was clean and the Nissl body was clear. Then dehydrated with 100% alcohol for 5 minutes × 2, transparented with xylene for 5 minutes × 2 and sealed with neutral gummi, and finally analysed under a microscope and collected images.

### 2.7 Golgi staining

After the rats (n = 3 for each group) being anaesthetized with 6% chloral hydrate (400 mg/kg), the aorta of the rat was inserted into with blunt infusion needle and perfused with 350‐500 mL (37°C) physiological saline containing 0.5% sodium nitrite into the systemic circulation quickly. When the liver became pale and the rinse become clear, continued to perfuse with fixed liquid containing 4% formaldehyde (500 mL) for about 1‐2 hours until the rat body was stiff. Then replaced the fixed liquid with mordant dyeing (500 mL, containing 5% chloral hydrate, 5% potassium dichromate, 4% formaldehyde), continued to pursue quickly for 5‐10 minutes to replace the systemic circulation stationary liquid. When flowing out thick orange liquid, slowed down the speed to 25 drop/minutes and the perfusion process of mordant dyeing liquid lasted about 3‐4 hours. The brain tissue after perfusion was taken and divided the brain into 2 parts along with the midline incision, kept the tissue containing hippocampus to 5 mm thick. Then dipped the tissue into fresh mordant dye, avoided light for 4 days and replaced the mordant dye every day. Next permutated the mordant dye with 1.5% silver nitrate solution for 3 days and avoided light and replaced a fresh silver nitrate solution daily. The brain slice (35 μm) was prepared by oscillation microtome (Germany, VT1000S, Leica). The slices were soaked in 2% potassium dichromate solution for 20 minutes and then rinsed with steaming water to clear. Then pasted the slices in 1.5% gelatine solution, dried dustproof air and then dehydrated with gradient alcohol, transparent with xylene and sealed piece with neutral gum. Finally, images were collected under a microscope to analyse. The spine numbers were measured by Image J software, and the different types of spines were analysed according to the schematic structure of the dendritic spines.^44^

### 2.8 Immunofluorescence staining

The brain sections were obtained as the immunohistochemistry staining described. The sections were incubated for 48 hours at 4°C with the first primary antibodies after being ruptured membrane and blocked with 5% BSA and then washed with PBS and incubated the second primary antibodies like described above. The sections were subsequently incubated with Rhodamine Green 488 or Red 546‐conjugated secondary antibodies (1:500) for 1 hour at 37°C. Pasted the brain sections to the glass slide and sealed them with 50% glycerine after being washed with PBS. The images were observed by a laser confocal microscope (LSM710, Zeiss, Germany), and the fluorescence images were analysed by the software affiliated.^45^, ^46^

### 2.9 Statistical analysis

Data were expressed as means ± SEM and analysed using SPSS 12.0 statistical software (SPSS Inc., Chicago, Illinois, USA). Means were compared by one‐way analysis of variance (ANOVA) procedure followed by LSD's post hoc Bonferroni's tests. *P* values <.05 were considered as significant.

## 3 RESULTS

### 3.1 TM induces UPR independent of GSK‐3 activation and causes tau hyperphosphorylation with spatial memory deficits in rats

**Figure 1:**
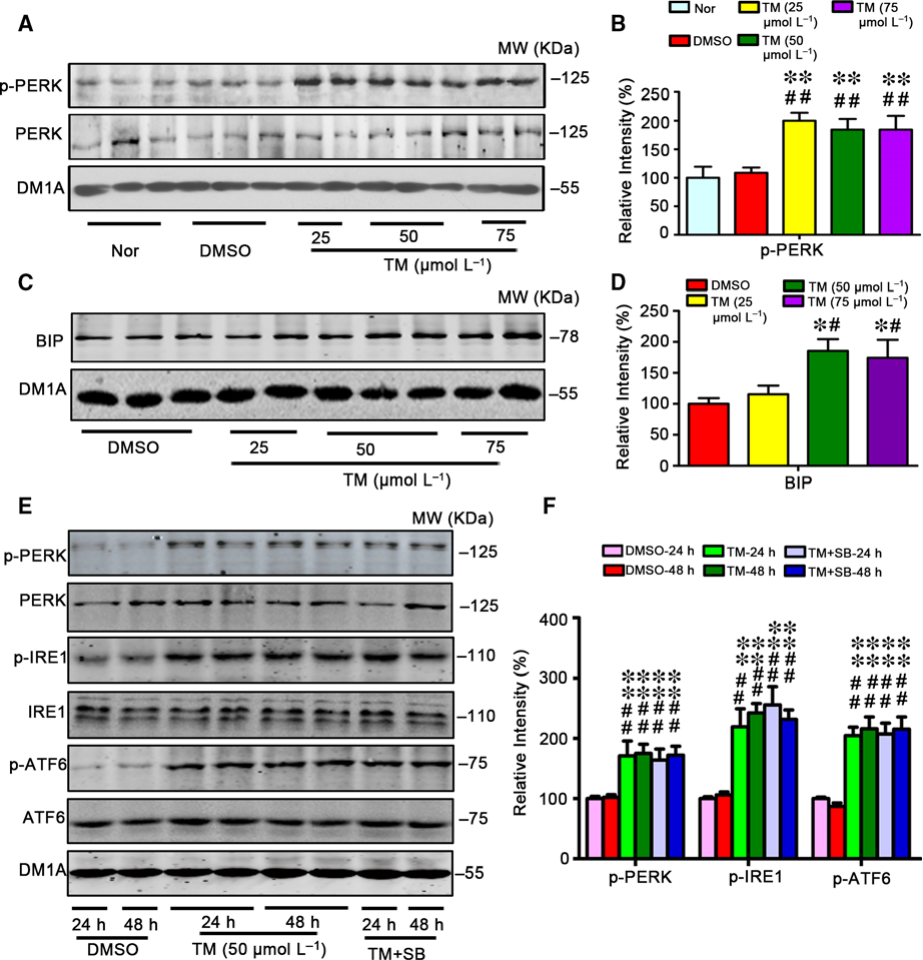
Tunicamycin induces UPR independent of GSK‐3 in rats. The male SD rats (4 m old) received ventricular infusion of 25, 50 or 75 μmol/L tunicamycin (TM, 10 μL) for 24 h (A,C), or infused with 50 μmol/L SB for 24 h and 48 h (E). The same volume of DMSO was infused as vehicle control, and the normal group (Nor) was killed without any treatment. The hippocampal extract was used for Western blotting (A,C,E) and quantitative analysis (B,D,F). The levels of the phosphorylated ER stress marker proteins as labelled except Bip were normalized against the total level, the latter and Bip were normalized against tubulin probed by DM1A. The data were expressed as means ± SD (n = 6). ** *P* < .01 vs Nor, ^##^ *P* < .01 vs DMSO in B; * *P* < .05 vs DMSO, ^#^ *P* < .05 vs TM (25 μm) in D; ** *P* < .01 vs DMSO‐24 h, ^##^ *P* < .01 vs DMSO‐48 h in F

To produce an in vivo UPR model, we infused different concentrations of ER stressor, TM, into the lateral ventricle of rats and measured the alterations of ER transmembrane protein, phosphorylated PERK (pPERK). We observed that infusion of TM at 25 μmol/L, 50 μmol/L and 75 μmol/L increased the protein level of pPERK, an ER stress marker (Figure 1A,B). Simultaneously, we found that level of Bip, an important ER‐associated chaperon, significantly increased by TM at 50 μmol/L and 75 μmol/L but not at 25 μmol/L (Figure 1C,D). Then, we infused the rats with 50 μmol/L of TM and measured the UPR, including pPERK, phosphorylated IRE‐1 (pIRE‐1) and phosphorylated ATF‐6 (pATF‐6) at different time‐points. The increased levels of pPERK, pIRE‐1 and pATF‐6 were detected at both 24 hours and 48 hours after the infusion (Figure 1E,F). In our previous study, we observed that TM could activate GSK‐3β. Therefore, we studied whether simultaneous inhibition of GSK‐3 by SB216763 (SB) affects UPR. The results showed that application of SB did not rescue UPR (Figure 1E,F). These results suggest that ventricular infusion of TM can induce UPR in rat brain independent of GSK‐3 activation.

**Figure 2:**
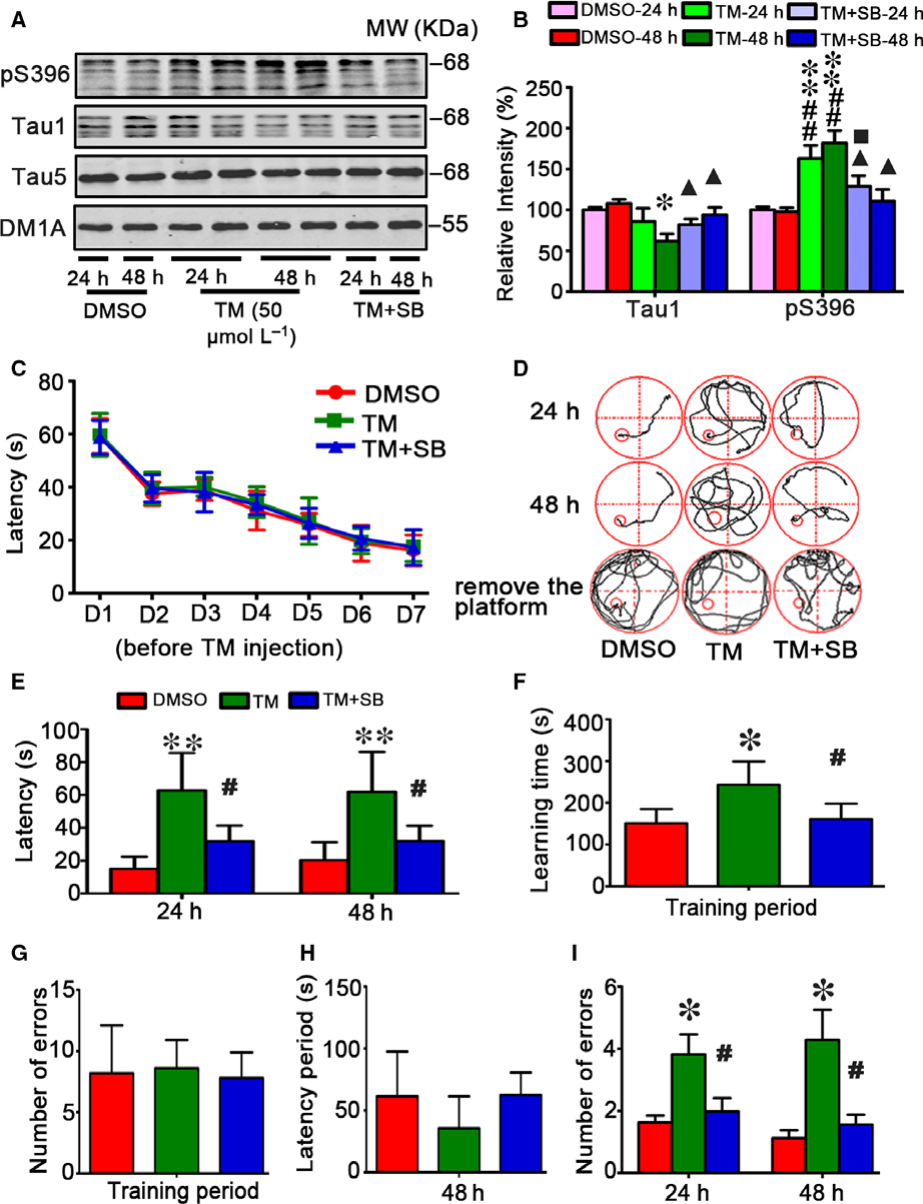
SB attenuates tau hyperphosphorylation and memory deficits induced by tunicamycin in rats. The rats were randomly divided into 3 groups infused, respectively, through ventricle with 50 μmol/L TM or DMSO or TM plus SB (50 μmol/L). The rats were trained in Morris water maze (MWM) for 7 days before DMSO, TM and TM + SB injection (C). After 24 or 48 h, the brain extract from hippocampal regions (HP) was used to measure the alterations of tau proteins by Western blotting (A) and quantitative analysis (B). The levels of unphosphorylated tau at Tau1 epitope and the phosphorylation level of tau at Ser396 epitope as labelled on the blot were normalized against total tau probed by Tau5 which was normalized against DM1A (n = 6). SB could more obviously rescue the decreased Tau1 and the increased phosphorylation level of tau at Ser396 epitope after TM being injected for 48 h. Simultaneously, the MWM and step‐down avoidance tests were used to assess learning and memory capacities (D‐I). The rats had same cognitive levels during 7 days training before TM treatment (C), while injection of TM for 24 or 48 h induced memory deficits shown by the increased latency to find the hidden platform in MWM test (D,E). TM‐injected rats used more time to learn to protect themselves from the risk of electric shock in the training period during step‐down avoidance test measured at 24 and 48 h after the injection (F). TM‐injected rats showed no difference of the number of errors compared with the control group rats (G). TM‐injected rats showed no difference of latency period but increased number of errors in the detection period during step‐down avoidance test measured at 24 and 48 h after the injection. SB rescued TM‐induced memory deficits shown by the significantly decreased latency to find the hidden platform in MWM test (E). The data were expressed as mean ± SD (n = 10). * *P* < .05, ** *P* < .01 vs DMSO‐24 h; ^#^ *P <* .05, ^##^ *P* < .01 vs DMSO **‐**48 h; ^■^ *P <* .05*vs* TM‐24 h, ^▲^ *P <* .05 vs TM‐48 h in B; * *P* < .05, ** *P* < .01 vs DMSO; ^#^ *P <* .05 vs TM in E‐I

In our previous studies, we found that TM treatment increased phosphorylated level of tau at Thr205, Thr231 and Ser396.^9^ We observed the similar alternation of tau proteins in this study and SB attenuated tau phosphorylation (Figure 2A,B). To measure the effects of UPR in spatial memory, we trained the rats for 7 consecutive days to allow remembering the hidden platform in water maze (Figure 2C), then we injected 50 μmol/L TM (10 μL) or isasteric DMSO or TM plus SB (50 μmol/L) into the rats lateral ventricle, after 24 or 48 hours, the hippocampus‐dependent spatial memory was measured by removed the platform. Compared with the DMSO‐injected control rats that could find the platform within 20 seconds by a direct searching strategy, while injection of TM increased the latency to about 60 seconds (Figure 2D,E). Learning and memory of the rats were also measured by step‐down avoidance tests. Compared with the DMSO vehicle control, TM treatment showed no difference of the number of errors in the training period. In the detection period during step‐down avoidance test, all the rats could not successfully avoid the risk of electric shock at 24 hours and there were no difference of latency period at 48 hours, but increased the number of errors both at 24 and 48 hours after TM injection (Figure 2F‐I). SB rescued TM‐induced memory deficits shown by the significantly decreased latency to find the hidden platform in MWM test and decreased the number of errors in step‐down avoidance tests (D‐I). These data suggest that TM can induce memory deficits of rats.

### 3.2 TM inhibits mushroom spine formation and expression of several synaptic proteins

**Figure 3:**
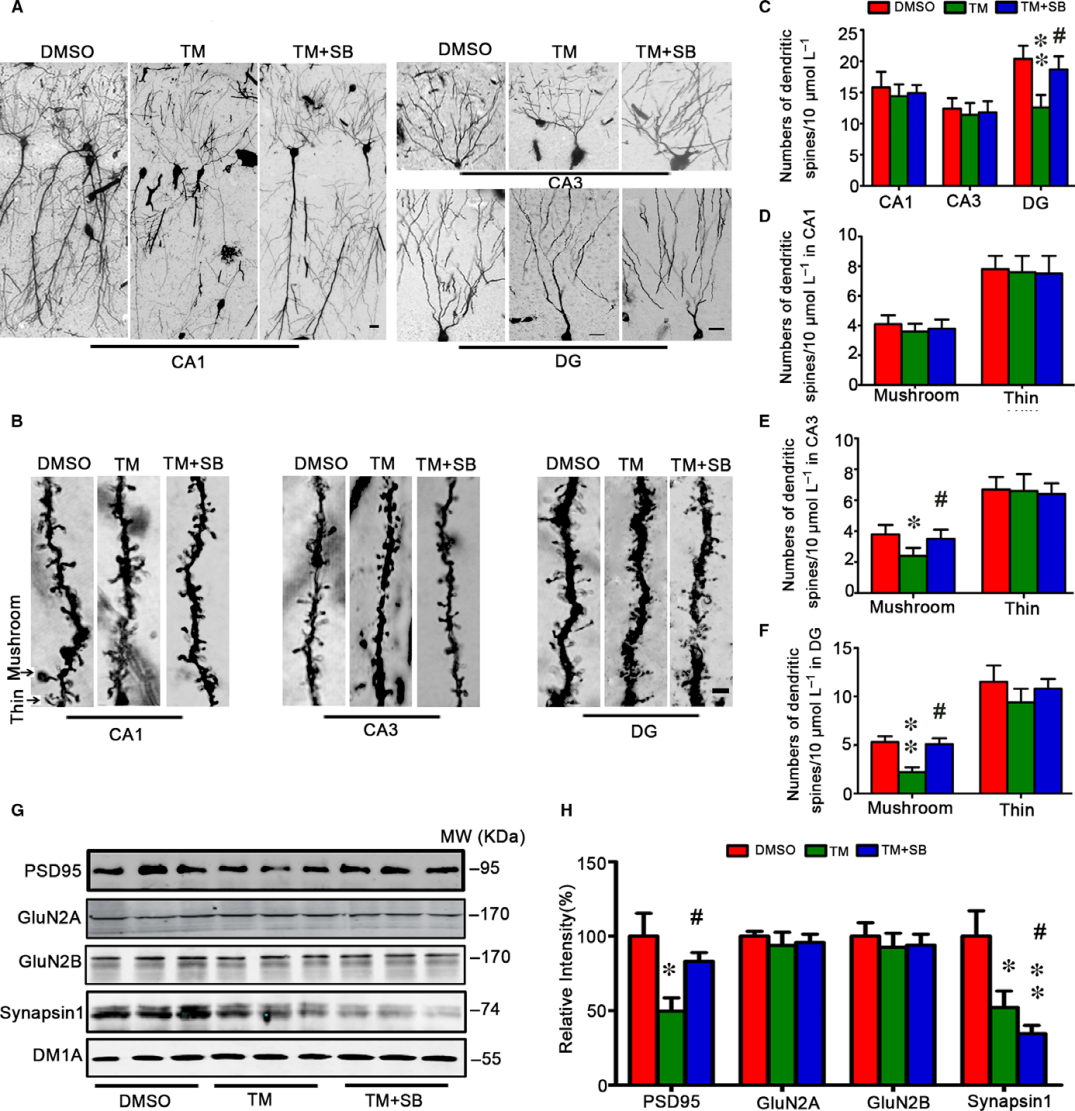
Tunicamycin inhibits mushroom spine formation and expression of synaptic protein and attenuation by SB. The representative images of dendritic spines in rat hippocampal CA1, CA3 and DG at 48 h after TM injection (A,B). TM decreased numbers of mushroom‐type spines significantly in DG and CA3 subsets but not in CA1 of the TM‐treated group, and no significant change of thin‐spines was detected (B‐F). SB could reverse the above phenomenon. The levels of synapse‐associated proteins were measured by Western blotting and quantitative analysis, normalized against tubulin probed by DM1A (G,H). The data were expressed as mean ± SD (n = 3 for A and B, bar = 50 μm for A, bar = 2 μm for B; n = 6 for G). * *P* < .05, ** *P* < .05 vs DMSO, ^#^ *P* < .05 vs TM

**Figure 4:**
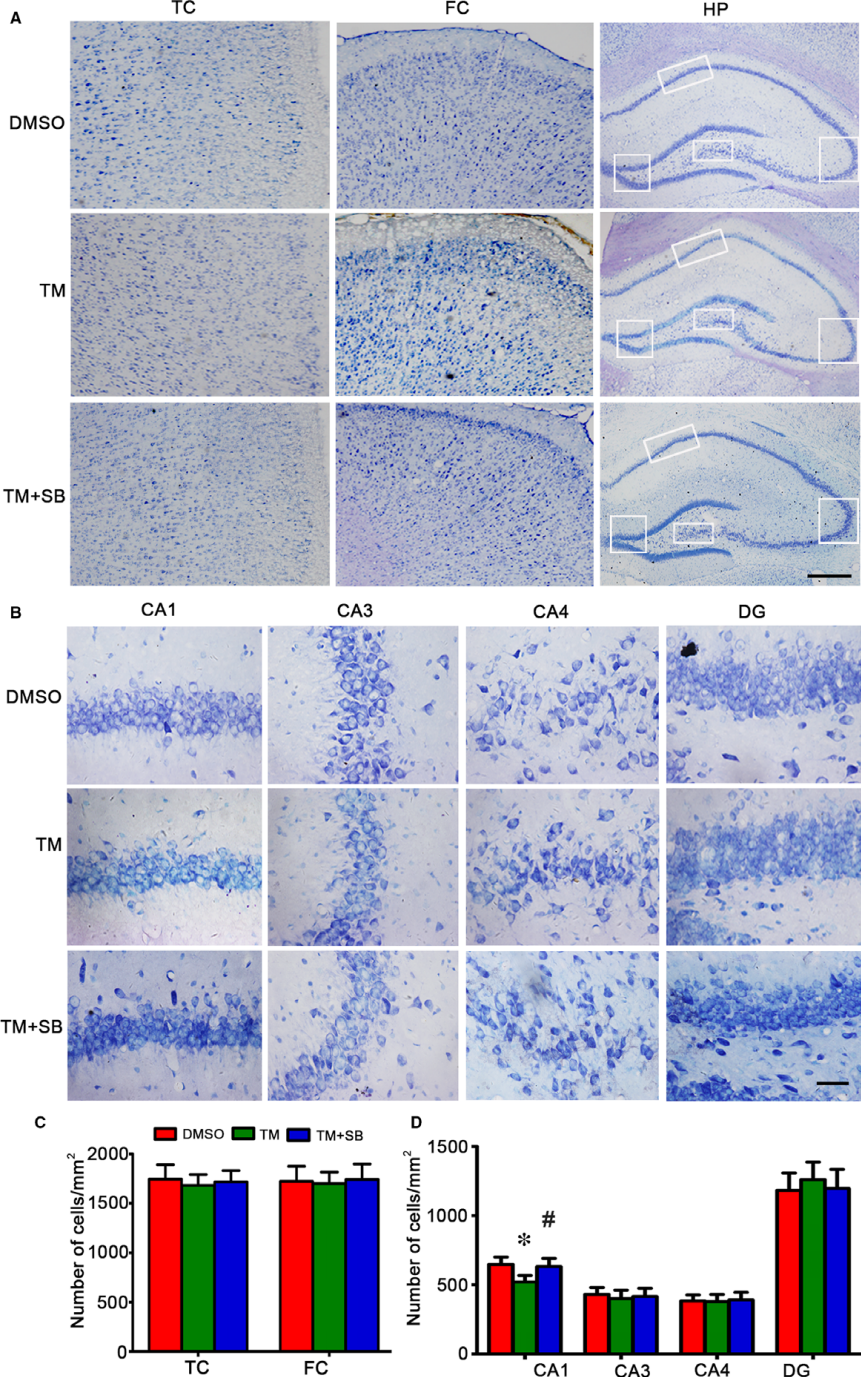
Tunicamycin induces cell loss in hippocampal CA1 subset and attenuation by SB. The representative Nissl staining analysis shows temporal cortex (TC), frontal cortex (FC) and hippocampus (HP) after TM injection for 48 h (A). The neuronal numbers in hippocampal CA1, CA3, CA4 and DG were analysed (bar = 500 μm for TC, FC and HP; bar = 50 μm for CA1, CA3, CA4 and DG) (B,C). The data were expressed as mean ± SD (n = 6). * *P* < .05 vs DMSO, ^#^ *P* < .05 vs TM

To explore the mechanisms underlying the TM‐induced spatial memory deficits, we measured spine morphology and synapse‐associated proteins. We found that number of mushroom‐type spines significantly decreased in DG (5.95 ± 1.02 vs 3.45 ± 0.98) and CA3 (3.85 ± 1.01 vs 2.42 ± 0.85) subsets but not in CA1 of the TM‐treated group, and no significant change of thin spines was detected (Figure 3A‐F). SB could reverse the decreased mushroom‐type spines in DG and CA3 subsets. We also measured the levels of synapse‐associated proteins. The results showed that levels of synapsin 1, a synaptic vesicle protein regulating pre‐synaptic release of glutamate, and the postsynaptic associated proteins, PSD95 significantly decreased in TM group, but GluN2A and GluN2B were no obvious alteration after TM injection (Figure 3G,H). These data suggest that TM induces impairments in hippocampal synaptic maturation. SB treatment restored PSD95 but not synapsin 1, instead it reduced level of synapsin 1. By Nissle's staining, we observed that cell number in hippocampal CA1 significantly decreased in TM group compared with control group, the decrease was not seen in DG subset, suggesting that TM induces cell death in CA1 subset and SB could reverse the cell death in CA1 subset (Figure 4A‐D).

### 3.3 TM treatment affects CREB phosphorylation with involvement of GSK‐3

**Figure 5:**
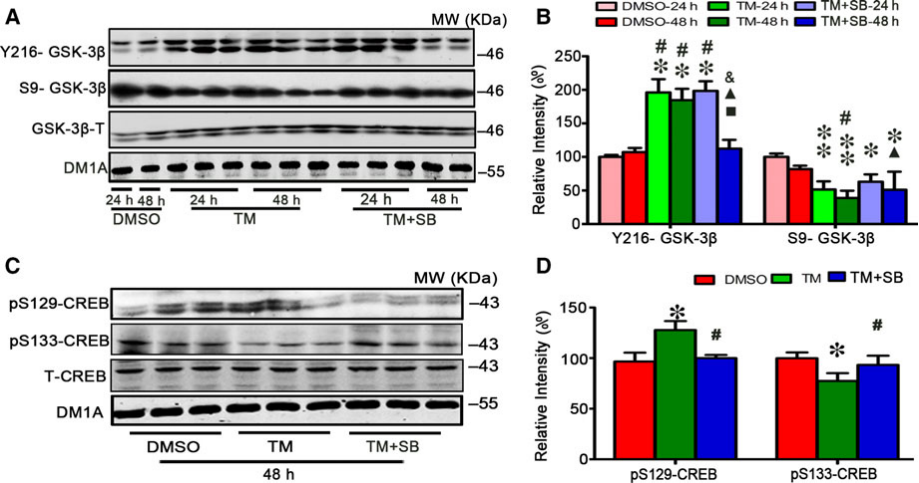
Tunicamycin treatment affects GSK‐3 and CREB phosphorylation. After ventricular infusion of DMSO, TM at concentration of 50 μmol/L or TM plus SB (SB, 50 μmol/L) for 24 and 48 h, GSK‐3β levels of the hippocampal extract were measured by Western blotting (A) and quantitative analysis (B). SB treatment rescued the increased Y216‐GSK‐3β and decreased S9‐GSK‐3β induced by TM especially at 48 h. And for 48 h, SB treatment restored the levels of pSer133‐CREB and pSer129‐CREB measured by Western blotting (C) and quantitative analysis (D). The data were expressed as means ± SD (n = 6) * *P* < .05, ** *P* < .01 vs DMSO‐24 h, ^#^ *P* < .05 vs DMSO‐48 h, ^■^ *P* < .05 vs TM‐24 h, ^▲^ *P* < .05 vs TM‐48 h, ^&^ *P* < .05 vs TM + SB‐24 h in B. * *P* < .05 vs DMSO, ^#^ *P* < .05 vs TM in D

To understand the mechanisms underlying the TM‐induced spatial memory deficit and altered synapse protein levels, we measured CREB, a crucial protein in regulating gene transcription. It was reported that GSK‐3 may be involved in CREB phosphorylation^47^; therefore, we measured GSK‐3β using activity‐dependent antibodies. We found that the active phosphorylation of GSK‐3β at Tyr216 (pY216‐GSK‐3β) increased and the inhibitory phosphorylation of GSK‐3β at Ser9 (pS9‐GSK‐3β) decreased at 24 and 48 hours after infusion of TM (Figure 5A,B), suggesting activation of GSK‐3β by TM. We observed that level of phosphorylated CREB at Ser133 (pS133‐CREB) was decreased in hippocampus treated with TM, while the level of phosphorylated CREB at Ser129 (pSer129‐CREB) was increased (Figure 5C,D). These alterations could be reversed by ventricular infusion of SB, the inhibitor of GSK‐3, especially at 48 hours (Figure 5A‐D). These data suggest that the alteration of CREB phosphorylation induced by TM may be related to the activation of GSK‐3.

**Figure 6:**
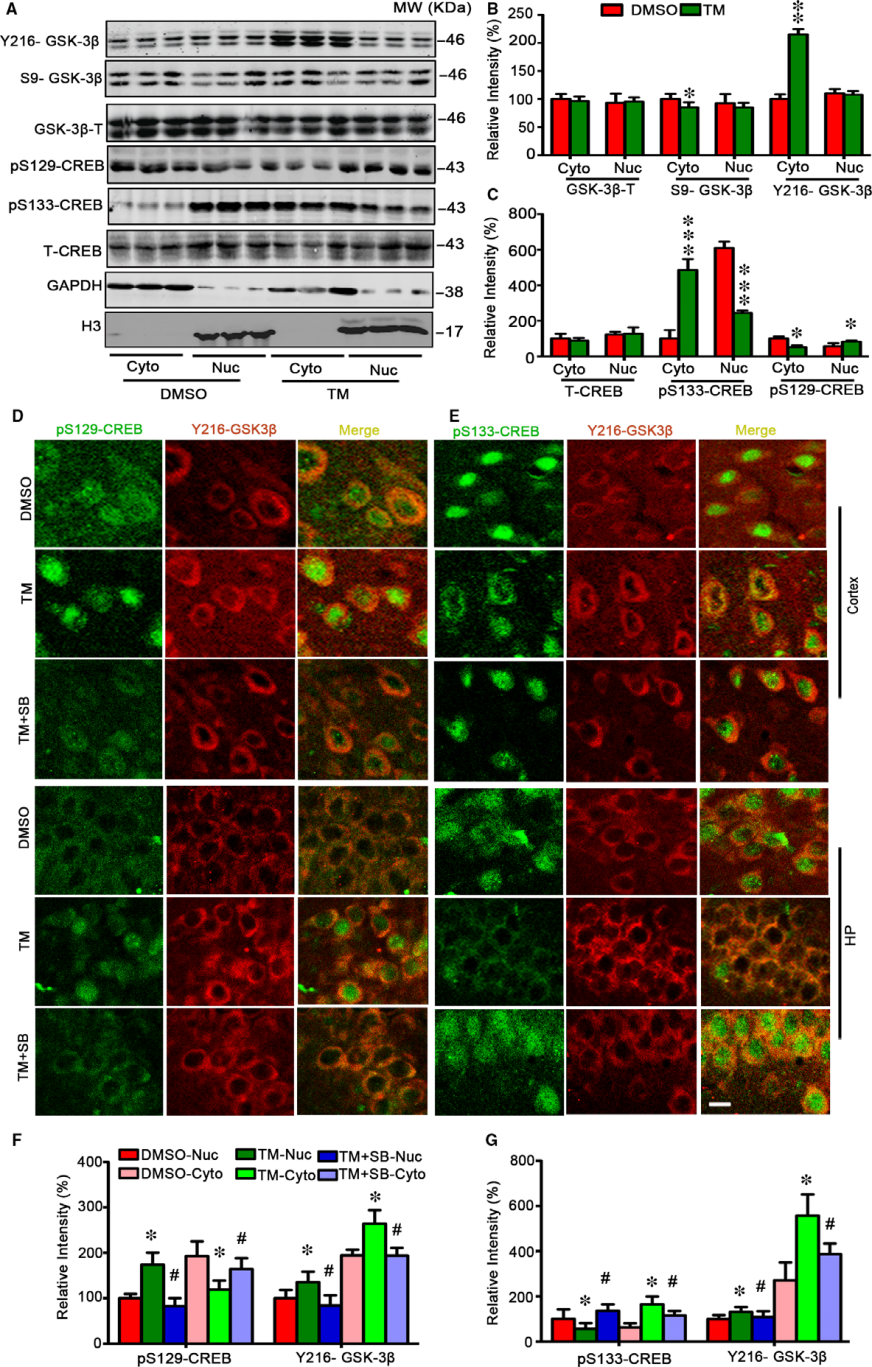
Tunicamycin increases nuclear co‐localization of Y216‐GSK‐3β and pSer129‐CREB with reduced pSer133‐CREB. Rats were treated with TM or DMSO for 48 h and then the cytoplasmic and nuclear fractions of the hippocampus were analysed by Western blotting and quantitative analysis (A‐C). The representative immunofluorescence images show phosphorylated CREB probed by pSer133 and pSer129 (green), and phosphorylated GSK‐3β by Y216‐GSK‐3β (red) in cortex and hippocampus (bar = 50 μm) (D,E). SB treatment restored the nuclear internal and external metastasis of pSer133‐CREB and pSer129‐CREB. The co‐localization of pY216‐GSK‐3β with p‐S129‐CREB and p‐S133‐CREB in hippocampus were statistically measured. The data were expressed as means ± SD. The tendency of pY216‐GSK‐3β with p‐S129‐CREB and p‐S133‐CREB in cortex was similar that in hippocampus (F,G) and the statistical graph not be showed. (n = 6) * *P* < .05, ** *P* < .01, *** *P* < .001 vs DMSO in B and C. * *P* < .05 vs DMSO *,* ^#^ *P <* .05 vs TM in F and G

To verify the role of GSK‐3β and CREB in TM‐induced spatial memory deficit and synapse impairments, we checked the levels of GSK‐3β and CREB both in cytoplasm fraction and in nuclear of hippocampus treated with TM. We observed that level of phosphorylated CREB at Ser133 (pS133‐CREB) was decreased in nuclear fraction and increased in cytoplasm of hippocampus treated with TM, while the level of phosphorylated CREB at Ser129 (pSer129‐CREB) in nucleus fraction was increased. Meanwhile, we observed that level of phosphorylated Y216‐GSK‐3β increased and level of phosphorylated S9‐GSK‐3β decreased in cytoplasm of hippocampus treated with TM (Figure 6A‐C). Immunofluorescence staining data also showed that level of pSer129‐CREB significantly increased in the nucleus of cortex and hippocampus and decreased in cytoplasm, while the level of pSer133‐CREB decreased in nucleus of hippocampus and increased in cytoplasm. Simultaneous inhibition of GSK‐3 by ventricular infusion of SB216763 rescued the alteration of CREB at pSer129‐CREB and pSer133‐CREB in nucleus fraction and cytoplasm of cortex and hippocampus treated with TM. An increased co‐localization of pY216‐GSK‐3β with pSer129‐CREB was also detected in the cytoplasm fraction (Figure 6D‐G). These data suggest that change of CREB phosphorylation induced by TM may be related to the activation of GSK‐3.

## 4 DISCUSSION

In the AD brains, the immunoreactivity of the ER stress markers, such as pPERK, eIF2α and IRE‐1α, was observed in hippocampal neurons associated with granulovacuolar degeneration, and the pPERK‐immunoreactive neurons were increased.^21^ Moreover, ER stress features are prominent in the brain of AD patients but not in Prion diseases,^22^ suggesting a specific role of ER stress in the pathophysiological process of AD. Many other evidence also suggests that ER dysfunction is closely related to AD. For instance, pPERK immunoreactivity was most abundant in the neurons with diffuse localization of the phosphorylated tau proteins.^22^ Exogenous Aβ can induce ER stress signalling pathways directly through Bip in cell culture. Furthermore, mutation in presenilin‐1 (PS1) gene is one of the most important factors of familial AD and the mutation of PS1 appears as unfolding protein in the ER. In addition, PS2 can be up‐regulated in sporadic familial AD which can inhibit UPR. These studies suggest a specific role of ER stress in the pathological process of AD.

In our recent studies, we observed that constant illumination could induce tau hyperphosphorylation, memory deficits and imbalance of kinases/phosphatases with ER damage.^48^ Rats brain treated with TM, an ER stress inducer, could significantly increase the phosphorylated tau.^9^, ^38^ These studies indicate a crucial role of ER stress in the AD‐like tau pathology and behavioural abnormalities. However, we are puzzled that whether and how ER stresses induce behavioural abnormalities. Whether the level of memory‐related kinase or other molecules, such as GSK‐3β or CREB, is also changed and what is the possible relation between them? Which parameter(s) is activated by ER stress and is responsible for the behavioural abnormalities?

To address these questions, we firstly produced an in vivo ER stress model by brain ventricular infusion of TM at different concentrations by measuring the increased ER transmembrane protein pPERK and Bip. Then, 50 μmol/L TM was selected to infuse rats for 24 and 48 hours, and elevation of 3 ER transmembrane proteins, including pPERK, pIRE‐1 and pATF‐6, was observed and the elevation was associated with memory deficits, suggesting that UPR could induce memory deficits in rats. When treated with TM plus SB, the levels of pPERK, pIRE‐1 and pATF‐6 had no alteration compared with the group treated with TM. These data indicate that inhibition of GSK‐3 by ventricular infusion of SB does not significantly affect TM‐induced UPR. Although TM treatment can induce ER stress with AD‐like tau hyperphosphorylation, we have to note that ER stress seen in the AD brains is a chronic process while TM treatment used in the current study is acute.

Tau is a major microtubule‐associated protein which stabilizes the neuronal cytoskeleton. Hyperphosphorylated tau which is incompetent in microtubules binding and stabilizing has reported to aggregate into filaments and ultimately lead to dysfunction of synapses, degeneration of neurons and cognitive impairment. Tau reduction that can block Aβ‐ and excitotoxin‐induced neuronal dysfunction has been represented to be an effective strategy for treating Alzheimer's disease and related conditions.^49^ In our previous study, we also demonstrated that UPR could induce increasing tau hyperphosphorylation in different brain regions.^9^ So we speculated the spatial memory deficit induced by TM was related to hyperphosphorylated tau. Simultaneously, we observed the number of neural cell in CA1 decreased after TM treatment, which suggests that the decrease of the neuron number in hippocampus CA1 may contribute to the learning and memory impairments induced by TM. Previous study has suggested that neurons in CA1 were more vulnerable to the stresses.^50^ Meanwhile, the dendritic spines in DG and CA3 subsets also decreased accompanied with the mushroom type of dendritic spines of hippocampus. The mushroom type of dendritic spines has been reported to be closely related to memory, and its plastic was limited. Moreover, the postsynaptic associated proteins, PSD95 and synapsin 1 significantly decreased after TM injection and SB could rescue the decrease in PSD95 but not synapsin 1; furthermore, it decreased the level of synapsin 1. The mechanism may deserve further investigation.

GSK‐3β is a key kinase that plays a crucial role in AD‐like tau hyperphosphorylation.^51^, ^52^ GSK‐3β activation or conditionally overexpressed GSK‐3β has been previously reported to cause spatial memory deficits in animals and inhibiting GSK‐3β could revise AD‐like cognitive deficit.^43^, ^53^, ^54^ An in vitro study also shows that GSK‐3β is activated during ER stress.^38^ However, it is still not understood whether and how GSK‐3β plays an in vivo role in ER stress‐induced tau phosphorylation and cognitive alterations. In this and our previous studies, we showed that UPR induced by TM and activated GSK‐3β that resulted in tau hyperphosphorylation in vivo and impaired spatial memory of rats. Inhibiting GSK‐3β by GSK‐3β inhibitor SB216763 could reverse the spatial memory detentions induced by ventricle brain injection TM.

In addition, studies proved that GSK‐3β could mediate phosphorylation of CREB, but its function and the mechanism was still not clear. Jason L. et al found that inhibitor of GSK‐3 could reduce the expression of fluorescein of RAW‐CRE cell treated with water toxin, which mediated by CREB. Simultaneously, they found water toxin did not activate the level of PKA‐dependent phosphorylation of CREB at Ser133 but increased the level of phosphorylation of CREB at Ser129.^55^ Some studies have shown that GSK‐3β could make CREB phosphorylate at Ser129. But there were conflicting views on CREB phosphorylation at Ser129. Some believe that phosphorylation at Ser129 causes the trans‐activation of GAL4‐CREB fusion protein and then promote the expression of syntrophic transcription factor and gene.^56^, ^57^ Some others reported that activating GSK‐3 and/or increasing the level of pS129‐CREB inhibit the transcription activity of CREB by decreasing the bonding affinity of CREB and DNA.^58^, ^59^, ^60^

In the present study, we found that TM could active GSK‐3β and increase the level of CREB phosphorylation at Ser129 in hippocampus, consequently resulted in a fall in learning and memory ability relevant CREB. At the same time, we found that TM could induce the increasing CREB phosphorylation at Ser129 locus in nucleus and CREB phosphorylation at Ser133 locus in cytoplasm of cortex and hippocampus, which maybe relate to CREB transfer both inside and outside the nuclear induced by TM and then regulate its downstream target genes.

Taken together, we find in the present study that TM‐induced UPR causes spatial memory deficits and synapse impairments with activation of GSK‐3. Simultaneous inhibition of GSK‐3 improves spatial memory and synaptic plasticity with mechanisms involving CREB phosphorylation at Ser129 and Ser133.
